# Friction and Wear Behaviors of PEEK/h-BN/SCF Composites Under Dry and Starved Oil Lubrications

**DOI:** 10.3390/ma19183966

**Published:** 2026-09-18

**Authors:** Zhe Tong, Xinghao Song, Wenxing Lei, Yajun Zhang, Jiaojiao Li, Li Zhao

**Affiliations:** 1 Shanxi Key Laboratory of Semiconductor Ultraprecision Machining Technology and Intelligent Equipment, North University of China, Taiyuan 030051, China; 2 School of Mechanical Engineering, North University of China, Taiyuan 030051, China; 3 Shan-Xi Jiang-Yang Chemical, Ltd., Taiyuan 030041, China; 4 School of Mechanical Engineering, North Minzu University, Yinchuan 750021, China

**Keywords:** PEEK composite, h-BN, SCFs, dry sliding, starved oil lubrications

## Abstract

The incorporation of various types of fillers is critical to enhancing the starved oil lubrication performance of PEEK matrix composites. In this work, PEEK composites reinforced with h-BN and SCFs were fabricated via a hot-pressing method, and the friction and wear behaviors under dry sliding and starved oil lubrication were systematically investigated. Surface oil wettability and Vickers hardness were characterized, and tribological tests were conducted on a ball-on-disk reciprocating tribometer. Worn surface topography and wear mechanisms were elucidated through laser scanning confocal microscopy and scanning electron microscopy. The results show that h-BN effectively reduces the friction coefficient through its solid lubrication effect, while SCFs significantly improve the hardness and load-bearing capacity of the PEEK matrix. Under dry sliding conditions, the PEEK composite containing h-BN and SCFs achieves the lowest wear rate of 10.3 × 10^−5^ mm^3^/(N·m), which is 74.6% lower than that of pure PEEK, indicating a synergistic anti-wear effect. Under starved oil lubrication, PEEK/h-BN maintains the smoothest and lowest friction, and the presence of SCFs further improves wear resistance of PEEK composites despite a slight increase in friction. In addition, the fillers’ incorporation reduces the surface oil wettability of the PEEK composites; the dominant wear mechanism hence transforms from severe adhesive wear and plastic deformation in pure PEEK to mild abrasive wear.

## 1. Introduction

Polyetheretherketone (PEEK) is increasingly adopted as a structural polymer to replace conventional metallic components in demanding engineering applications, such as aerospace actuators and radial piston hydraulic motors, owing to its exceptional mechanical strength, superior thermal stability and outstanding chemical inertness [1,2,3]. However, in practical industrial operations, friction pairs are frequently subjected to a severe and transient tribological condition, which is induced by start–stop cycles or reciprocating motions, wherein the liquid lubricant cannot sustain a continuous lubricant film [4,5,6]. Direct contact at the sliding interface leads to adhesive wear, localized stress concentrations, and extreme frictional flash temperatures at the interface, which induce rapid thermal softening of the polymer matrix and ultimately result in component failure [7,8]. Consequently, there is an urgent demand for enhancing the lubrication performance and wear resistance of PEEK composites.

The addition of carbon fibers is widely utilized to enhance the load-bearing capacity of the polymer matrix composites. Hintze et al. compared the tribological performance of CF-reinforced PEEK composites under both dry and liquid-lubricated sliding conditions, pointing out that while CFs significantly enhance load-bearing efficiency, the exposed rigid fibers act as abrasives against the counterface when the lubricant film ruptures [9]. Man et al. investigated the friction and wear behavior of additively manufactured continuous carbon fiber-reinforced polyamide 6 composites, revealing that fiber orientation critically governs subsurface crack propagation pathways [10]. Concurrently, Schroeder et al. mathematically categorized the microstructural failure modes in the sliding wear of PEEK matrix composites, identifying delamination and interfacial debonding as the dominant mechanisms under high contact pressures [11]. Hou et al. demonstrated that the broken lubricant film causes the exfoliated fiber to transform into third-body particles, thereby triggering severe furrow formation [12]. Overall, these hard fibrous materials can effectively optimize the friction reduction and wear resistance of PEEK matrix composites by regulating interface heat dissipation and stress distribution under diverse lubrication conditions [13,14,15,16].

In summary, carbon fiber reinforcement alone can effectively improve the wear resistance of PEEK composites under sufficient lubrication, but it cannot compensate for liquid film failure under starved lubrication due to the absence of a solid lubricating phase. Additionally, the exposed rigid fiber edges after lubricating film rupture induce exacerbated third-body abrasive wear, which further degrades tribological stability of the composites [17,18,19]. Two-dimensional (2D) materials with low interlayer shear strength and high in-plane strength can be extruded from polymer matrices to fill micro-asperity valleys on the surface of counterpart, which buffer the sharp carbon fiber edges and form boundary isolation layers to dynamically repair transfer films and compensate for fluid film failure under starved lubrication [20,21,22]. The structural integrity of boundary lubricating films under extreme contact pressures relies on micro-chemical reactions and physical adsorption, while localized frictional heat triggers cross-linking, spreading and substrate adhesion of polymer wear debris to promote stable transfer film formation [23,24,25].

The previous investigations mostly focused on dry sliding environments or fully liquid (oil/water) lubrication conditions [26,27,28]. However, the synergistic mechanism between hexagonal boron nitride (h-BN) and short carbon fibers (SCFs) during oil film rupture remains unclear. Therefore, this study aims to investigate the synergistic lubrication effect of h-BN- and SCF-reinforced PEEK composites under starved oil lubrication conditions, to reveal the solid–liquid interface lubrication mechanism of h-BN- and SCF-reinforced PEEK composites, and provide theoretical guidance for the design of reliable, long-service-life engineering tribological pairs.

## 2. Materials and Experiment

### 2.1. Materials

**Figure 1:**
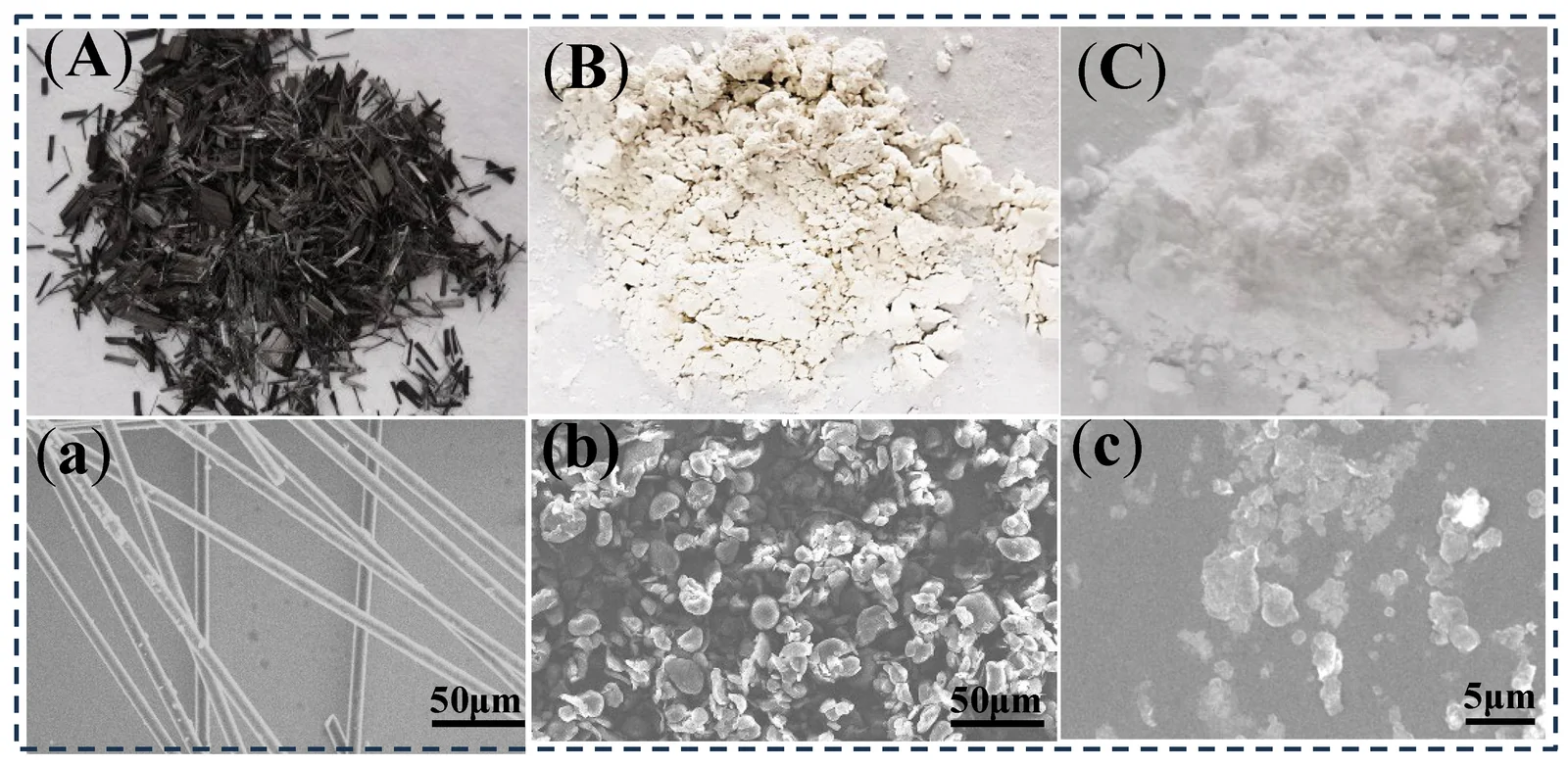
Microtopography of (**A**,**a**) SCFs, (**B**,**b**) PEEK powder and (**C**,**c**) h-BN particles.

SCFs with a diameter of 7 μm and length of 3–5 mm were supplied by Zhongfu Shenying Carbon Fiber Co., Ltd., Lianyungang, China (Figure 1A,a). PEEK powder (analytical reagents) with an average size of 20 μm was purchased from Jiangxi Fengtai New Materials Co., Ltd., Jiujiang, China (Figure 1B,b). h-BN particles (analytical reagents) with an average size of 2 μm were purchased from Aladdin Reagent (Shanghai) Co., Ltd., Shanghai, China (Figure 1C,c). Gear lubricant oil with a dynamic viscosity of 0.15 Pa·s and density of 0.87 kg/L (10W-40, China) was purchased from PetroChina Lubricant Company (Beijing, China). All chemical reagents were used as received without further purification.

### 2.2. Preparation of PEEK Composites

**Figure 2:**
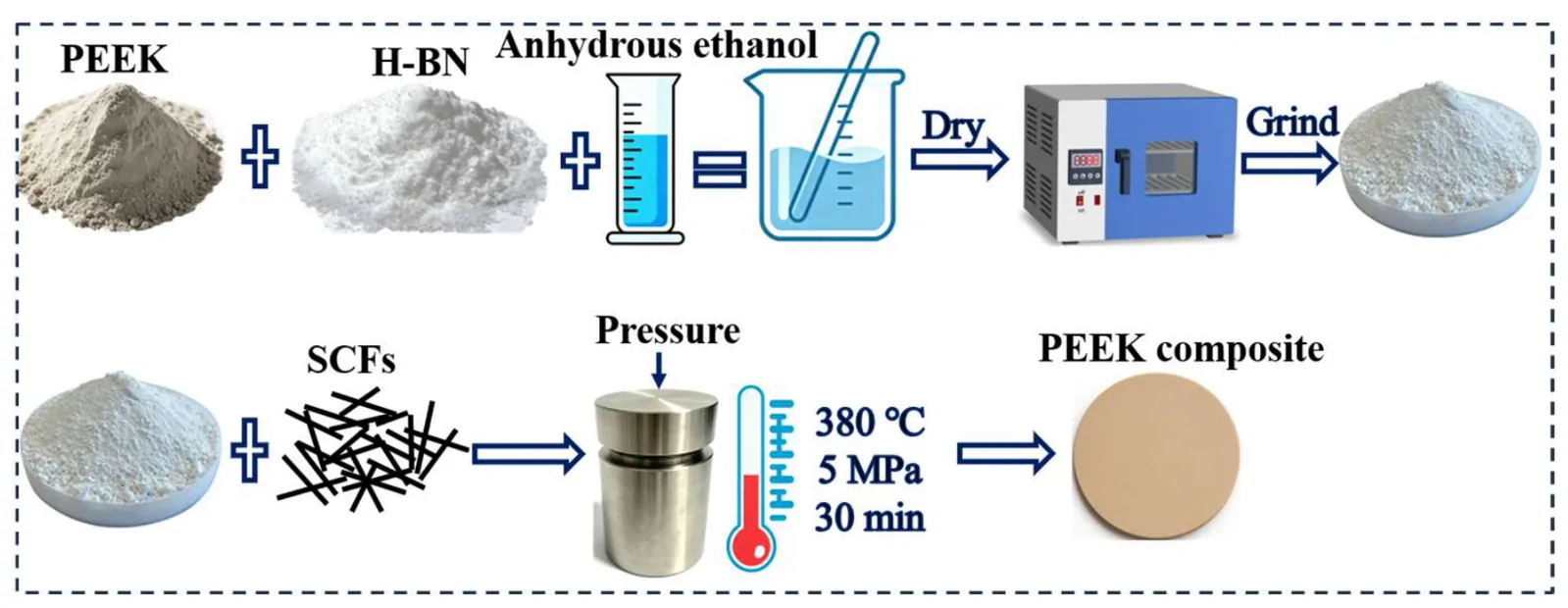
Schematic diagram of the preparation procedure of PEEK composites.

As shown in Figure 2, PEEK with a 10 wt.% of h-BN was dispersed in 20 mL of anhydrous ethanol, magnetically stirred for 30 min, and then subjected to ultrasonic treatment for 30 min. Next, the suspension was dried in a vacuum oven at 110 °C for 6 h to remove the solvent, followed by grinding to obtain a homogeneous powder mixture. Subsequently, the mixture of powder and SCFs was processed via high-shear mixing for 2 h. The resulting blended powder was transferred into a cylindrical mold with a size of *ϕ*30 mm × 10 mm. Finally, the mixture was hot-pressed at 380 °C under 5 MPa for 30 min and then cooled naturally to room temperature under ambient conditions to obtain the PEEK composites. The detailed compositions of PEEK composites are listed in Table 1.

### 2.3. Tribology Tests and Characterization Method

**Figure 3:**
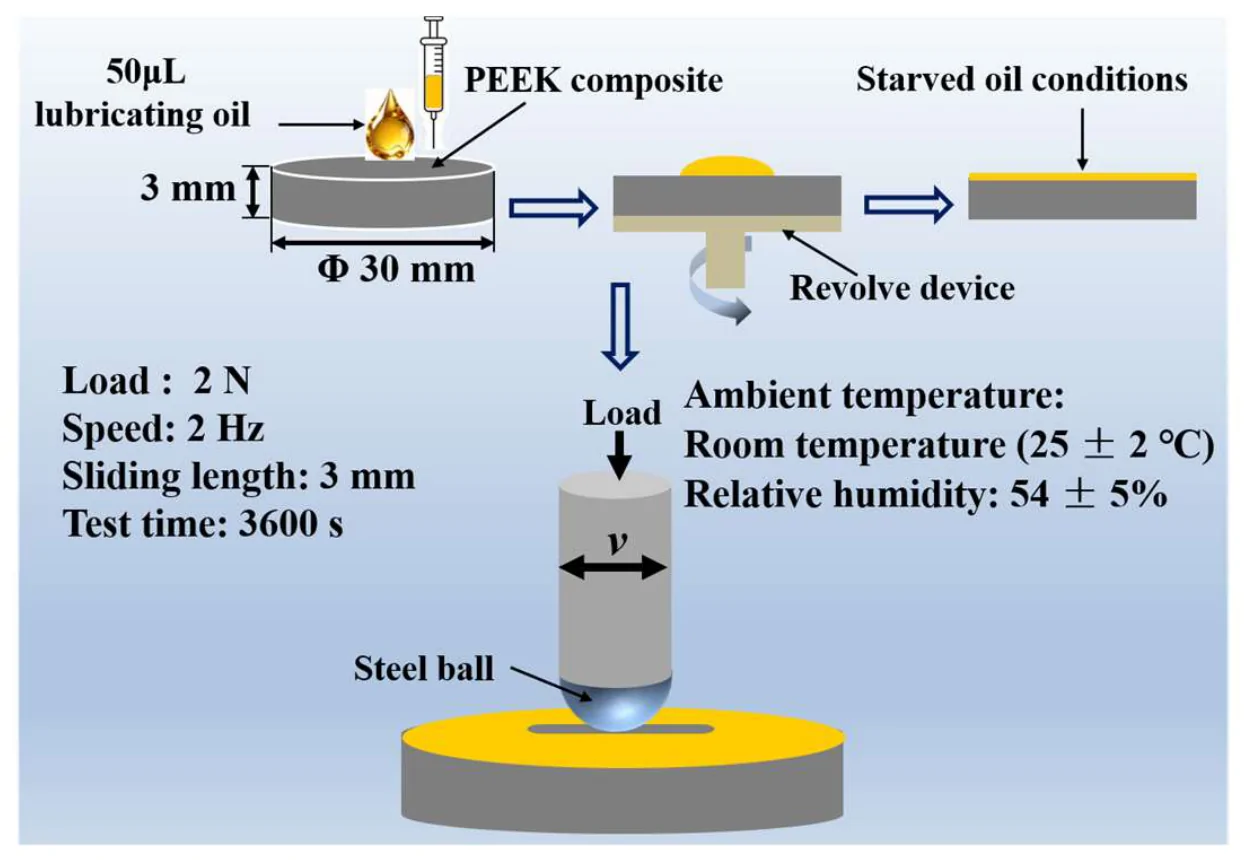
Schematic diagram of tribological characterization.

The tribological properties of PEEK composites were evaluated using a ball-on-disk reciprocating tribometer under starved oil and dry sliding conditions at room temperature; the test schematic diagram configuration is illustrated in Figure 3. The lubrication conditions are starved oil lubrication and dry sliding. For the starved oil friction test, the composites were mounted on a rotating platform, and 50 μL of gear oil was dispensed onto its central region. Subsequently, the platform was then rotated at a controlled speed to enable uniform distribution of the oil film across the specimen surface via centrifugal force, thereby establishing starved-lubrication conditions. A stainless steel ball with a diameter of 9.6 mm (Ra ≈ 0.22 μm) and a hardness of 60 ± 2 HRC served as counterpart. All tests were conducted under a normal load of 2 N and a sliding frequency of 2 Hz with a sliding length of 3 mm. All tribological tests were carried out under ambient conditions: a temperature of 25 ± 2 °C and relative humidity of 54 ± 5% RH. Every test was repeated three times.

The wear volume and wear track dimensions were measured and evaluated using a laser scanning confocal microscope (Olympus, Tokyo, Japan, OLS5000). The surface topography and microstructural features of the worn surfaces of the composites and the ball were further characterized by a scanning electron microscope (Ciqtek, Hefei, China, SEM3200 at 10 kV) and an optical microscope.

Contact angle measurements were performed using a dynamic contact angle measurement instrument to evaluate the oil wettability of the PEEK composites. For each composite, 10 measurements for each sample were performed and the average values were taken as results. The hardness of PEEK composites was measured by a Vickers hardness instrument (TIME, Beijing, China, TMVS-1) with an indenter load of 0.32 N, with each sample repeated 5 times for accuracy. The thermal conductivity of PEEK composites was measured by using a laser thermal conductivity meter (NETZSCH LFA 447, Selb, German) at room temperature.

## 3. Results and Discussion

### 3.1. Effects of h-BN and SCFs on the Hardness and Wettability of PEEK Composites

**Figure 4:**
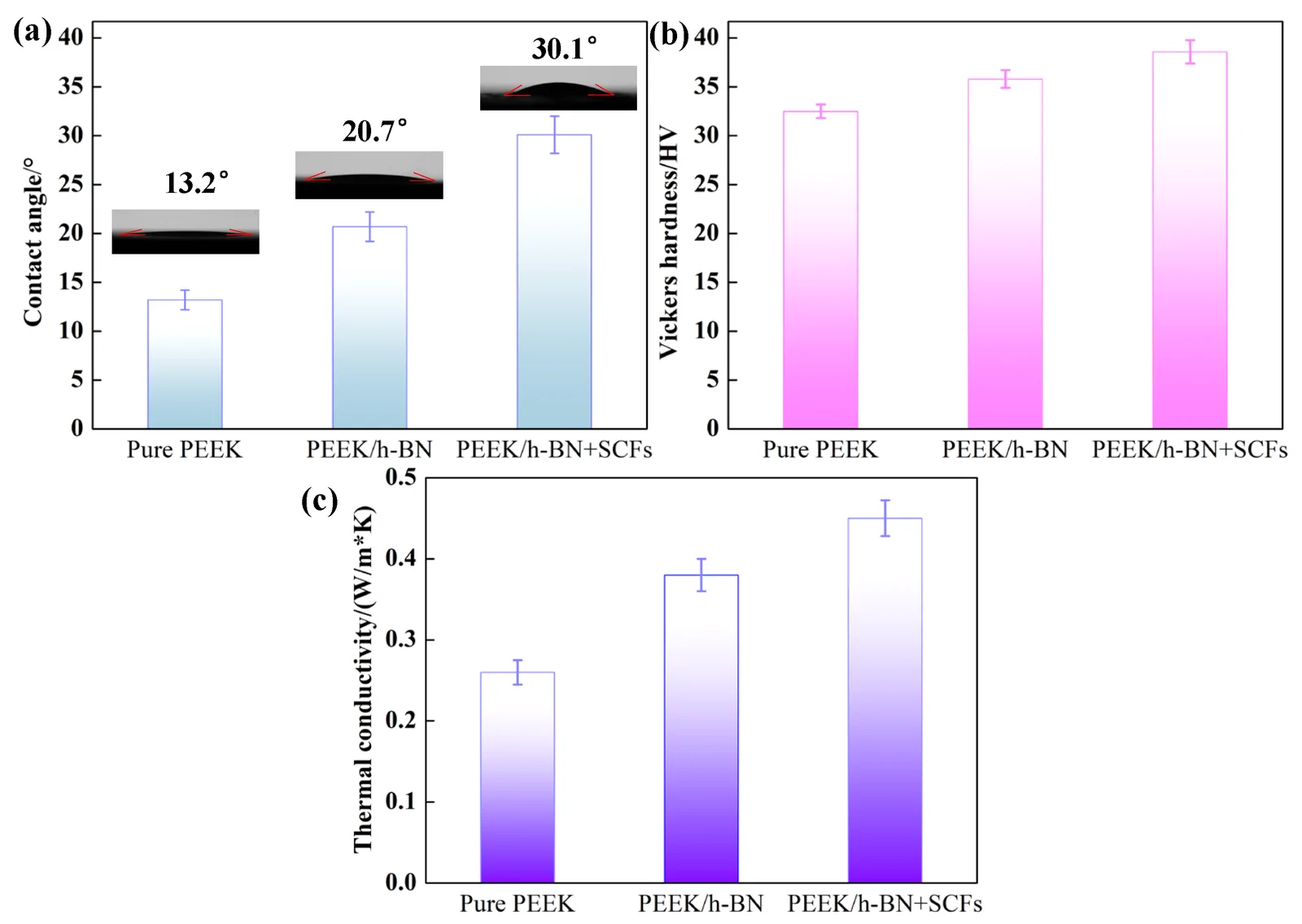
(**a**) Oil contact angle, (**b**) Vickers hardness and (**c**) thermal conductivity of the PEEK composites.

The oil contact angles of the PEEK composites are presented in Figure 4a. As shown, the contact angle increases markedly with the incorporation of h-BN and SCFs, indicating reduced oil wettability. This reduction in wettability arises from the synergistic interplay between decreased surface free energy and altered microstructural topography. Both h-BN and SCFs possess lower surface energy than the PEEK matrix, thereby elevating the intrinsic contact angle. Moreover, the co-incorporation of SCFs and h-BN generates a heterogeneous micro-nanoscale surface morphology, and air pockets become entrapped within interstitial voids among exposed filler particles, impeding oil droplet from entering surface grooves and consequently enhancing the macroscopic contact angle [29].

Figure 4b presents the Vickers hardness of PEEK composites reinforced with various fillers. As shown, pure PEEK shows a hardness of 32.5 HV. Furthermore, with the incorporation of h-BN, the hardness of the PEEK composite increased by 10.2% compared to that of pure PEEK. With the subsequent addition of SCFs, the hardness reached 38.6 HV, marking an 18.8% increase compared to pure PEEK. This improvement is primarily attributed to the SCFs and h-BN with high tensile modulus and strength, which effectively absorb stress and restrict the plastic deformation of polymer chains.

It can be seen that pure PEEK exhibits a thermal conductivity of 0.26 W·m^−1^·K^−1^ from Figure 4c. And the thermal conductivity increased to 0.38 W·m^−1^·K^−1^ with the incorporation of h-BN, corresponding to a 46.2% enhancement relative to pure PEEK. With further addition of SCFs, the thermal conductivity reaches 0.45 W·m^−1^·K^−1^. This trend confirms that the hybrid filler system effectively constructs interconnected thermally conductive pathways within the PEEK matrix.

### 3.2. Tribological Properties of PEEK Composites Under Dry Sliding Conditions

**Figure 5:**
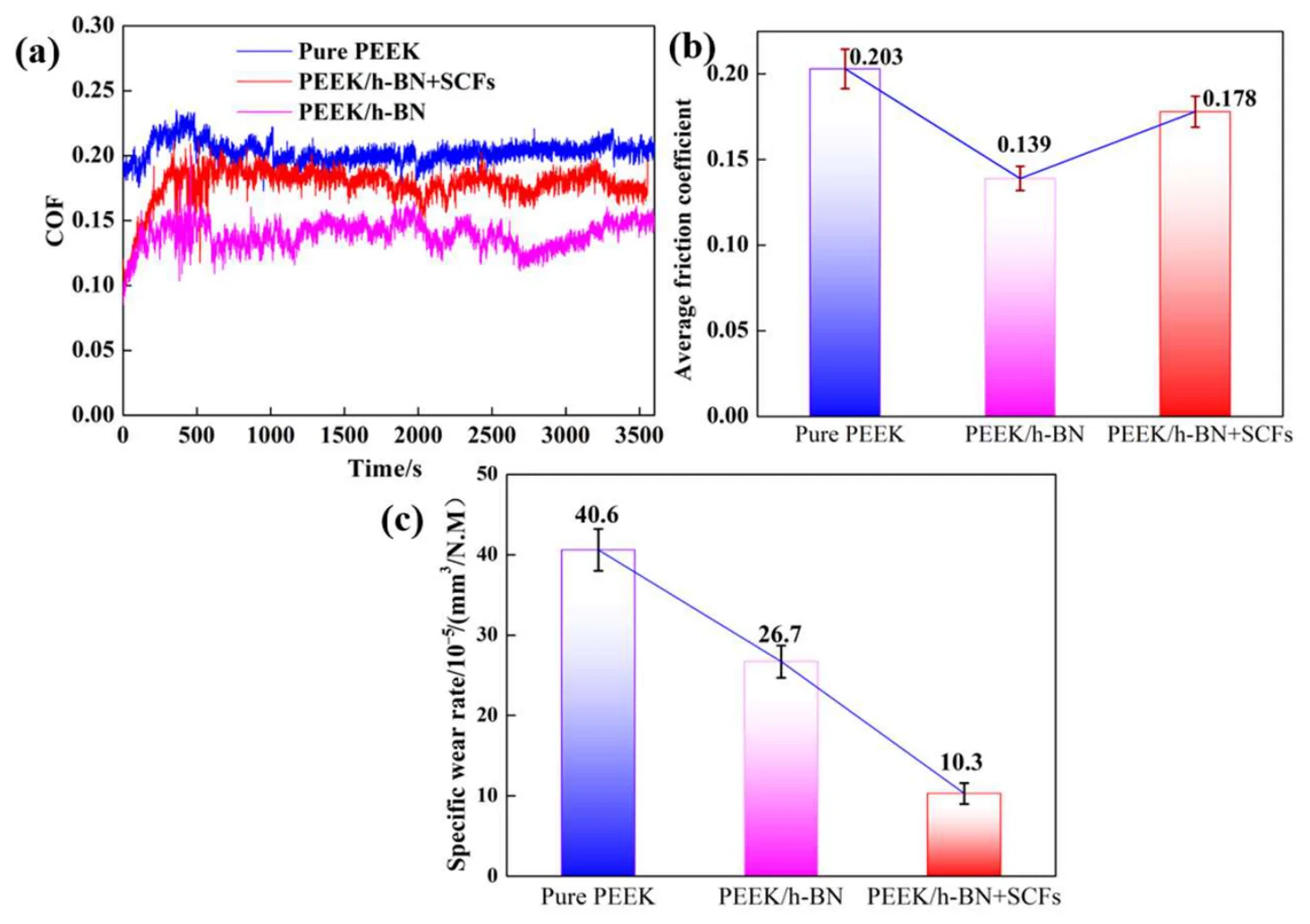
Tribological properties of PEEK composites under dry sliding conditions: (**a**) real-time coefficient of friction; (**b**) average friction coefficient; (**c**) specific wear rate.

Figure 5 shows the tribological performances of three types of PEEK composites under dry sliding conditions. As described in Figure 5a, all samples exhibit a distinct running-in period during the initial 500 s, characterized by a rapid rise in COF followed by the transition to a relatively stable steady-state sliding stage. Pure PEEK displays the highest COF, which fluctuates around 0.20, with a fluctuation range from approximately 0.18 to 0.23 throughout the whole sliding process. In contrast, the PEEK/h-BN composite achieves the lowest real-time COF, stabilizing within 0.13 to 0.15 after the running-in phase. Meanwhile, the COF curve shows the smallest fluctuation amplitude, indicating a smoother and stable sliding state. However, with the addition of SCFs, PEEK/h-BN/SCF exhibits reduced lubrication performance. The COF fluctuates between 0.16 and 0.19, which is lower than that of pure PEEK but higher than that of PEEK/h-BN, indicating that SCFs may have disrupted the formation and performance of the transfer film due to their high rigidity [30].

As shown in Figure 5b, the average COF of pure PEEK is 0.203. After incorporating h-BN, the average COF of PEEK/h-BN decreased to 0.139, corresponding to a reduction of nearly 31.5% relative to pure PEEK. With the further introduction of SCFs, the average COF of PEEK/h-BN/SCFs rises to 0.178. Figure 5c presents the specific wear rates of the three types of PEEK composites. Pure PEEK exhibits the highest specific wear rate of 40.6 × 10^−5^ mm^3^/(N·m), indicating the lowest wear resistance. The addition of h-BN reduces the wear rate of the PEEK composite to 26.7 × 10^−5^ mm^3^/(N·m), achieving a 34.2% decrease compared with pure PEEK. Notably, PEEK/h-BN/SCF exhibits the optimal wear resistance, with a specific wear rate of 10.3 × 10^−5^ mm^3^/(N·m), which represents an approximately 74.6% reduction compared with pure PEEK, and a 61.4% reduction relative to PEEK/h-BN.

**Figure 6:**
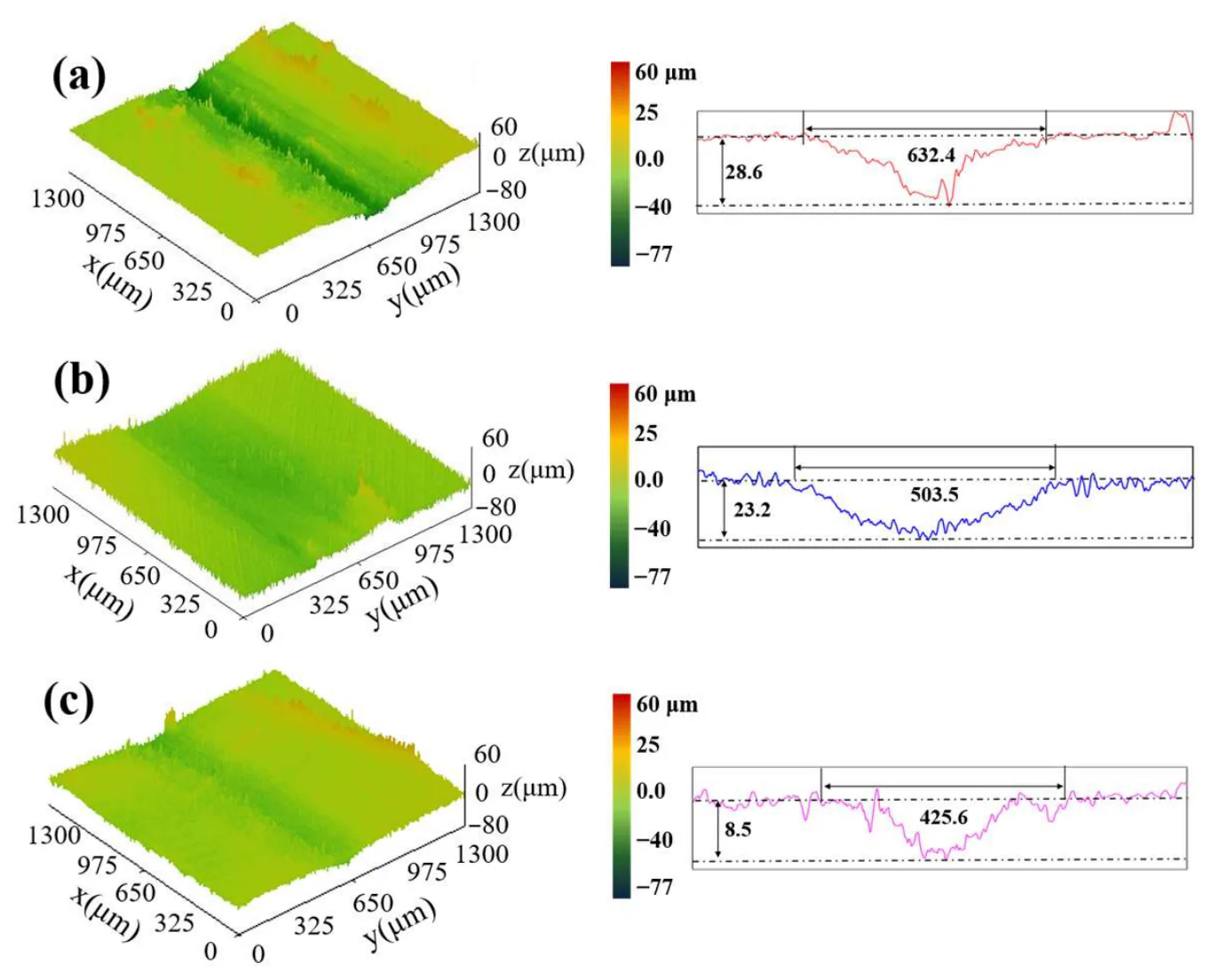
3D topographies and scratch profiles of PEEK composites under dry sliding conditions: (**a**) pure PEEK; (**b**) PEEK/h-BN; (**c**) PEEK/h-BN/SCFs.

Figure 6 presents the 3-D worn surface topographies and corresponding cross-sectional profiles of three types of PEEK composites under dry sliding conditions. For pure PEEK (Figure 6a), a wide and deep continuous wear scar is observed on the 3D topography accompanied by extensive plastic deformation, resulting in high surface roughness. The cross-sectional profile shows a wear scar width of 632.4 μm and a maximum depth of 28.6 μm, indicating substantial material removal and the lowest wear resistance. The h-BN-reinforced PEEK composite exhibits significantly reduced wear scar width and depth compared to pure PEEK (Figure 6b). The wear scar width is 503.5 μm, with a depth of 23.2 μm, corresponding to reductions of approximately 20.4% and 18.9% relative to pure PEEK respectively, confirming that h-BN effectively enhances the wear resistance of the PEEK matrix. For PEEK/h-BN/SCF (Figure 6c), the wear scar is further narrowed and shallowed, the cross-sectional profile shows a width of 425.6 μm and a depth of 8.5 μm, which are 32.7% and 70.3% lower relative to pure PEEK respectively, indicating that the improvement in wear resistance is attributable to the synergistic reinforcement of h-BN and SCFs.

**Figure 7:**
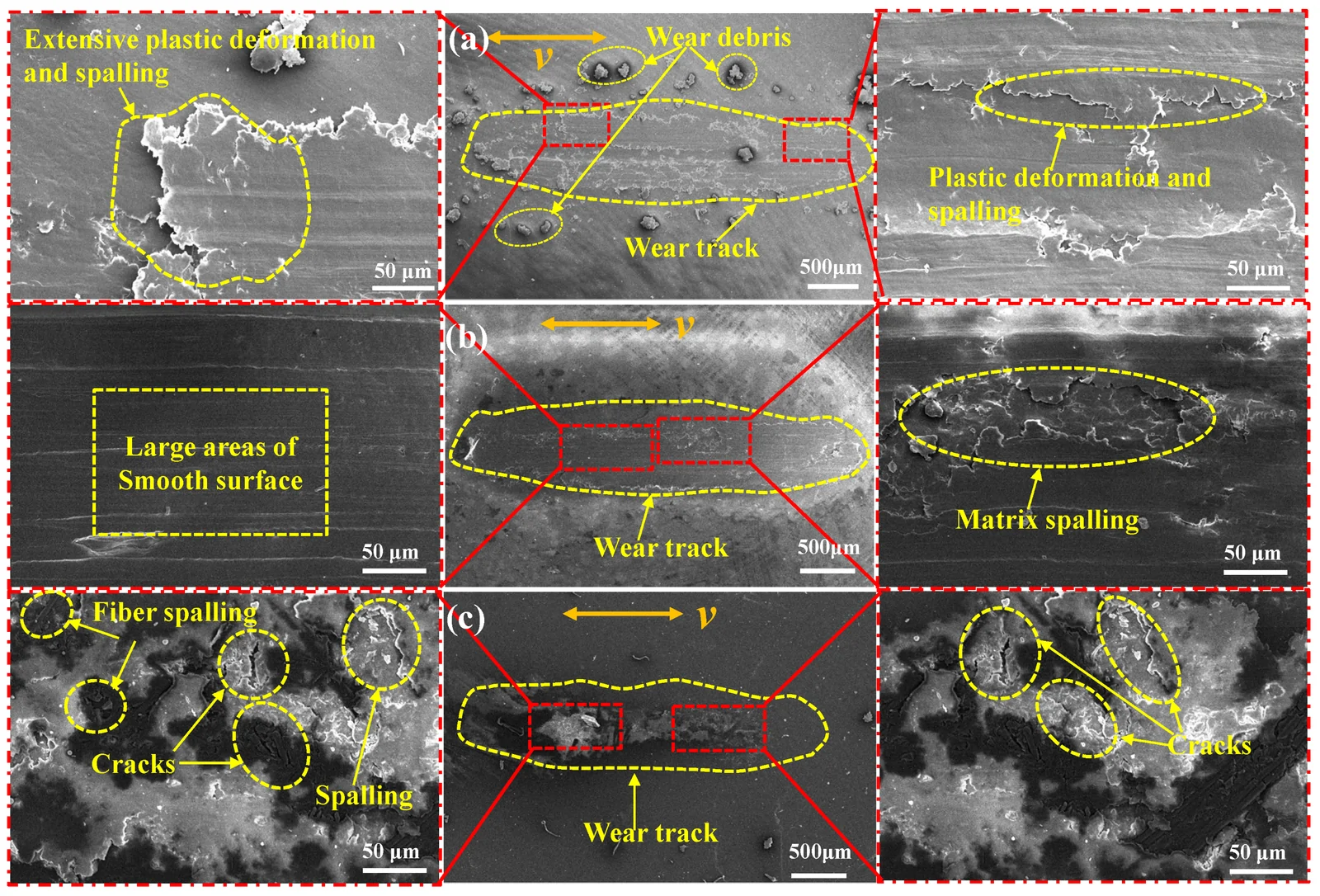
Morphology of worn surfaces of PEEK composites under dry sliding conditions: (**a**) pure PEEK; (**b**) PEEK/h-BN; (**c**) PEEK/h-BN/SCFs.

Figure 7 presents the worn surface morphology of PEEK composites under dry sliding conditions. As shown in Figure 7a, pure PEEK exhibits a wide, irregular wear track with a large number of granular and flaky wear debris dispersed along the sliding direction, indicating severe material removal. The worn surface is characterized by extensive plastic deformation and large-scale matrix spalling. Owing to the low thermal conductivity and insufficient load-bearing capacity of the PEEK matrix, frictional heat accumulated at the sliding interface cannot be dissipated efficiently, which leads to thermal softening of the PEEK matrix and a dramatic drop in shear resistance. The softened surface then undergoes plastic extrusion and delamination under combined normal loading and tangential shear stress, resulting in uneven deformation and irregular subsurface spallation pits. Concurrently, detached wear debris becomes entrapped at the asperity contacts and acts as abrasive particles, further accelerating wear progression. The dominant wear mechanisms are severe adhesive wear and plastic deformation. In contrast, the PEEK/h-BN (Figure 7b) displays a narrower and smoother wear track with reduced wear debris generation. A large region of relatively flat worn surface is observed, featuring only minor matrix spalling and shallow parallel scratches. As a 2D material with high thermal conductivity, h-BN can not only accelerate the dissipation of interfacial heat and inhibit the thermal softening of the PEEK matrix, but also facilitate the formation of a continuous and uniform transfer film on the surface of its counterpart [31]. Consequently, the dominant wear mechanism shifts from severe adhesion/plastic flow to mild abrasive and adhesive wear, and the specific wear rate is substantially reduced relative to pure PEEK.

The h-BN/SCF-reinforced PEEK composite (Figure 7c) exhibits the narrowest wear track among all PEEK composites. Only microcracks and local matrix spalling occurred on the worn surface, and the damages are confined to the shallow surface layer. The wear resistance of PEEK/h-BN/SCFs can be attributed to the synergistic effect of SCFs and h-BN. SCFs with high strength and modulus significantly enhance the hardness, elastic modulus and load-bearing capacity of the PEEK matrix, thereby effectively resisting compressive and shear forces and suppressing large-scale plastic deformation and adhesive delamination. The microcracks and limited matrix spalling are mainly attributed to the stress concentration at the fiber–matrix interface under cyclic friction loading, leading to mild interfacial debonding and matrix spalling [32,33].

### 3.3. Tribological Properties of PEEK Composites Under Starved Oil Conditions

**Figure 8:**
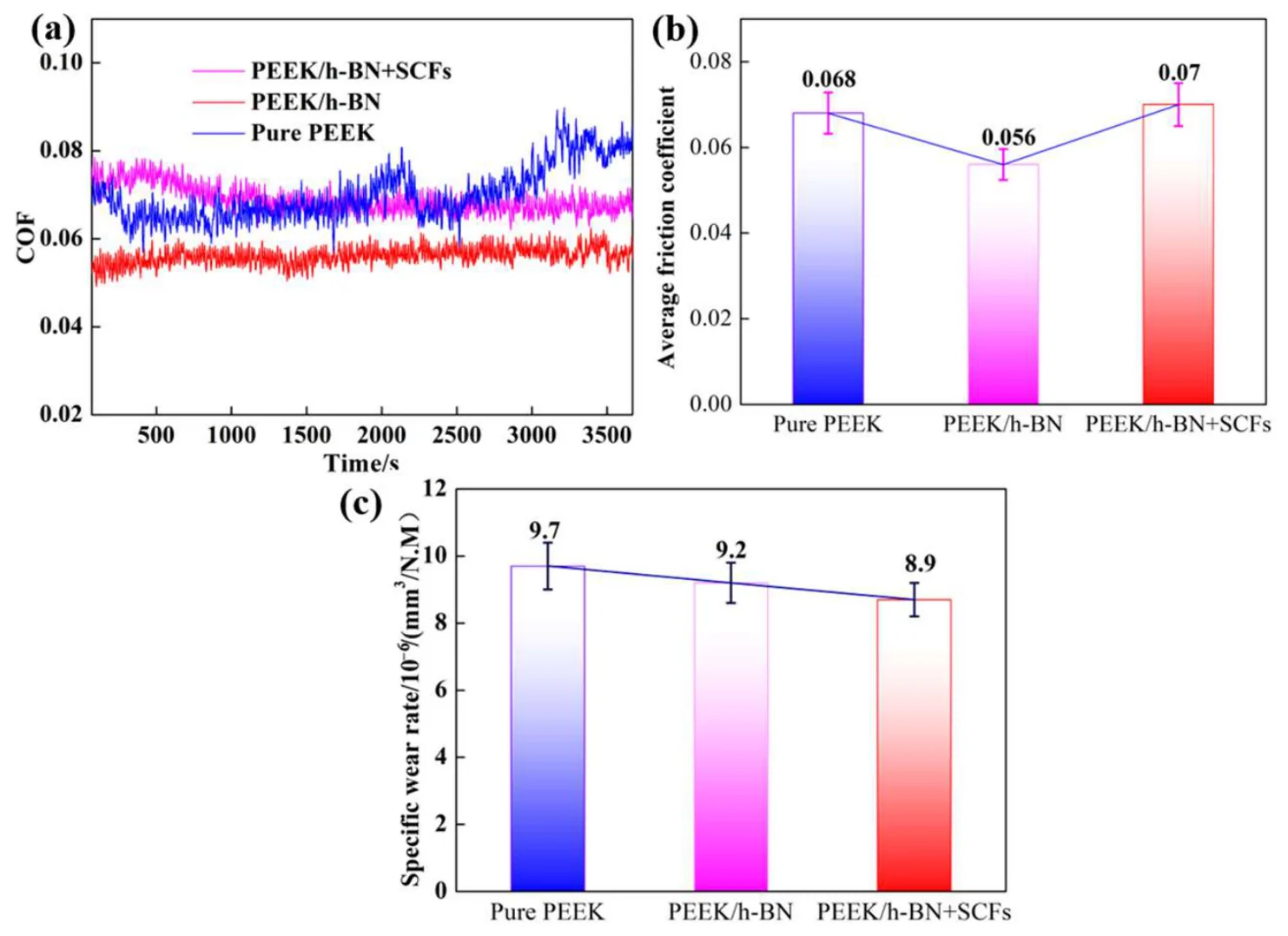
Tribological properties of PEEK composites under starved oil lubrication: (**a**) real-time coefficient of friction; (**b**) average friction coefficient; (**c**) specific wear rate.

Figure 8 illustrates the tribological performances of PEEK composites under starved oil lubrication conditions. As shown in Figure 8a,b, all three composites exhibit a brief running-in period. Pure PEEK displays the highest and most fluctuating COF, ranging from 0.06 to 0.09, indicating unstable interfacial contact under insufficient lubrication. PEEK/h-BN exhibits the lowest and smoothest COF curve at approximately 0.055. After adding SCFs, the COF of PEEK composites increases slightly relative to PEEK/h-BN. This rise is primarily attributed to the non-layered crystalline structure of SCFs increasing direct abrasive contact with the steel counterface, thereby partially offsetting the lubricating effect of h-BN [34,35]. As presented in Figure 8c, the specific wear rate decreases from 9.7 × 10^−6^ mm^3^/(N·m) for pure PEEK to 9.2 × 10^−6^ mm^3^/(N·m) for PEEK/h-BN, reaching a minimum of 8.9 × 10^−6^ mm^3^/(N·m) for PEEK/h-BN/SCFs. Although SCFs do not further reduce friction, their high load-bearing capacity effectively suppresses plastic deformation and delamination under normal loading. These results demonstrate the competitive and synergistic effects of h-BN and SCFs in governing the tribological behavior of PEEK composites under starved oil lubrication.

**Figure 9:**
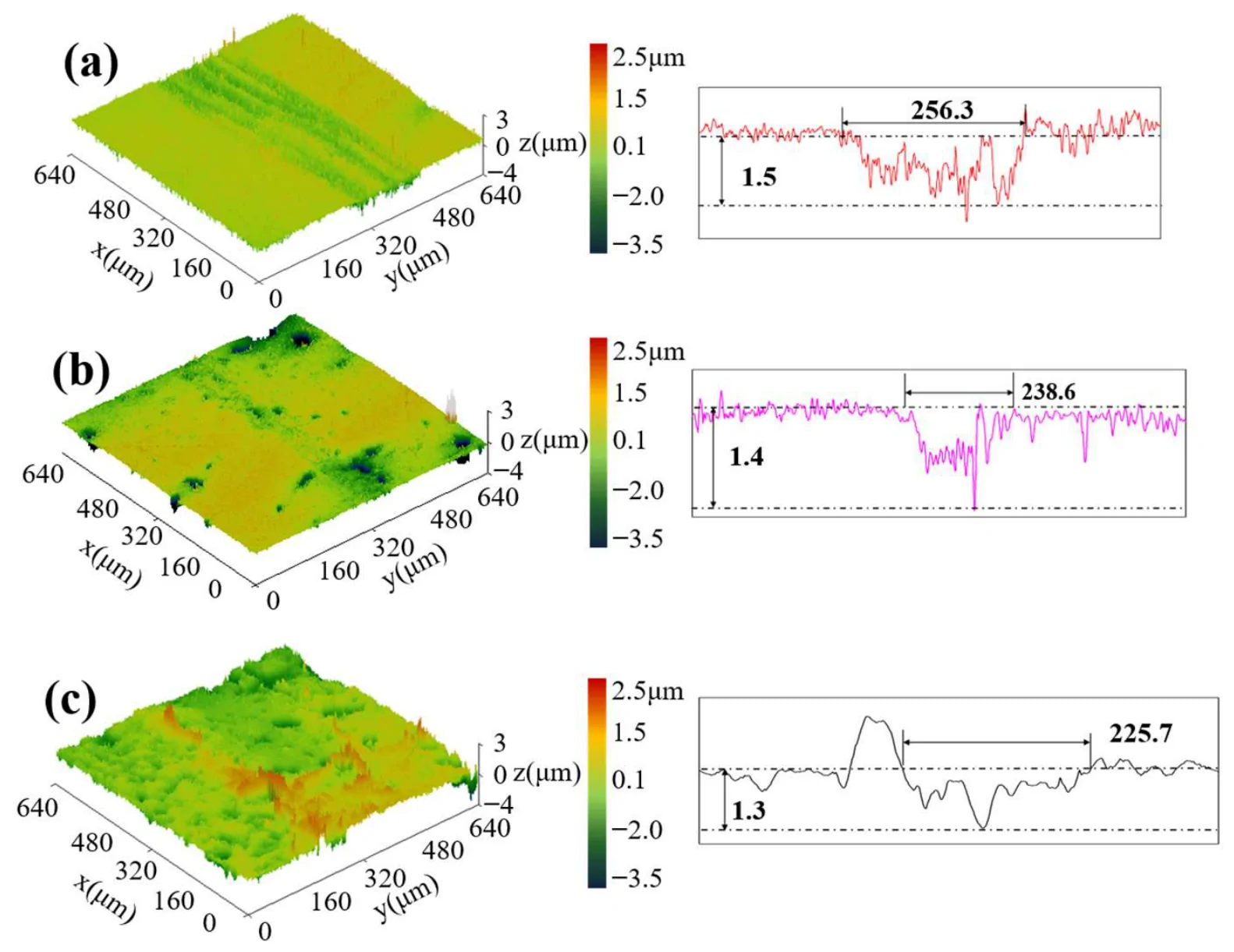
3D topographies and scratch profiles of PEEK composites under starved oil lubrication: (**a**) pure PEEK; (**b**) PEEK/h-BN; (**c**) PEEK/h-BN/SCFs.

Figure 9 presents the 3D worn surface morphologies and corresponding cross-sectional wear track profiles of three types of PEEK composites under starved-lubrication conditions. For pure PEEK (Figure 9a), the worn surface is dominated by plowing grooves parallel to the sliding direction, forming a wear track with a width of 256.3 μm and a maximum depth of approximately 1.5 μm. In contrast, PEEK/h-BN (Figure 9b) exhibits a narrower and shallower wear track instead of prominent continuous plowing furrows, indicating that the addition of h-BN modifies the surface failure mode without significantly altering the wear volume. For the PEEK/h-BN/SCF composite (Figure 9c), the wear track width and depth are 225.7 μm and 1.3 μm respectively, showing a slight decrease compared to the former two composites. Notably, its entire surface displays substantially higher micro-roughness and undulations, which originates from the inherent presence of micron-scale SCFs; the fiber ends constitute numerous micro-asperities on the surface. The change in roughness directly affects the friction coefficient of the PEEK composites [36].

**Figure 10:**
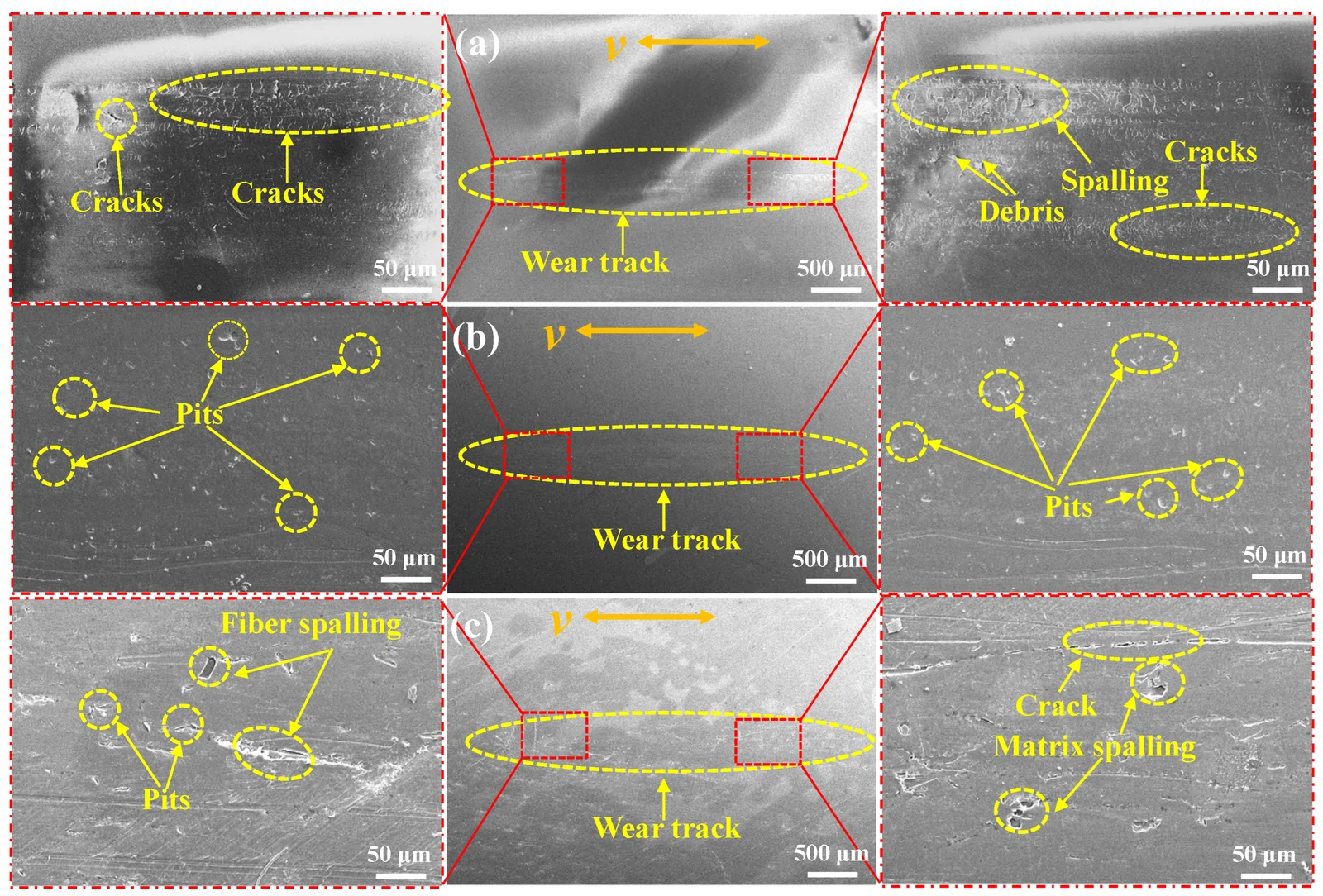
Morphologies of worn surfaces of PEEK composites under starved oil conditions: (**a**) pure PEEK; (**b**) PEEK/h-BN; (**c**) PEEK/h-BN/SCFs.

The worn surface morphologies of PEEK composites under starved oil conditions were analyzed, and the results are shown in Figure 10. Clearly, the distinct wear morphologies are observed at different locations within the scratch region of pure PEEK. The left zone is dominated by crack propagation and localized delamination, whereas the right region exhibits more extensive debris formation, the appearance of cracks and matrix spalling. This phenomenon is primarily attributed to the non-uniform distribution of the lubricant oil film under extreme starvation conditions, leading to localized dry friction zones. In contrast, PEEK/h-BN exhibits markedly uniform wear morphology across the entire scratch track due to the synergistic effect of solid lubrication and boundary lubrication. The worn surface is characterized by the appearance of micro-pits. After the incorporation of SCFs, stress concentration induced by non-conformal contact under point-contact conditions gives rise to stress mismatch at the sliding interface between carbon fibers and the PEEK matrix, leading to crack formation consistent with the morphology observed on the surface of PEEK/h-BN/SCFs [37,38].

### 3.4. Tribological Mechanism

**Figure 11:**
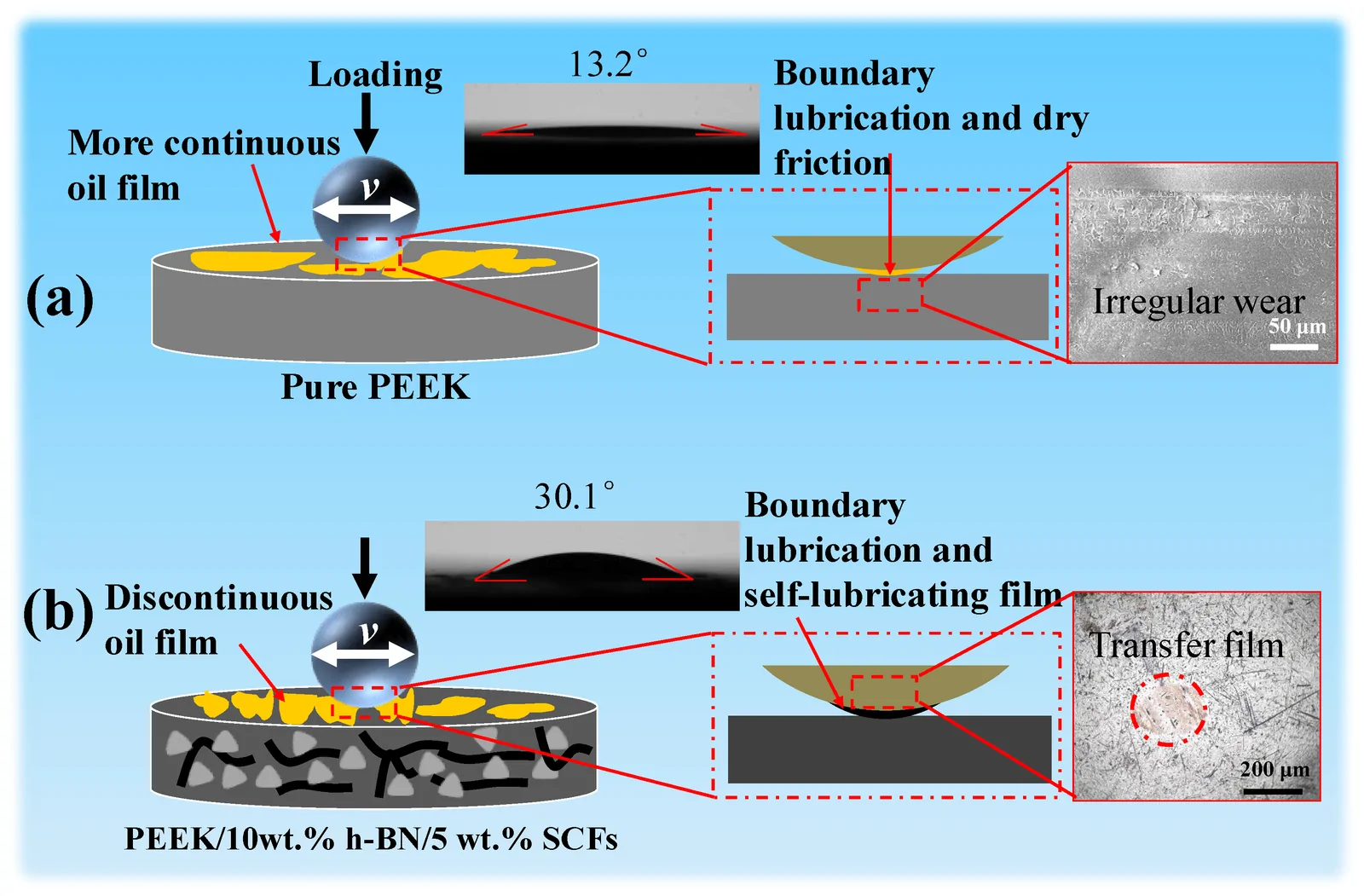
Lubrication and anti-wear mechanism of PEEK composites reinforced by h-BN and SCFs under starved oil lubrication: (**a**) pure PEEK; (**b**) PEEK/h-BN/SCFs.

Based on the above friction and wear results and analysis, the lubrication and wear mechanisms of h-BN and SCFs acting on the tribological properties of PEEK matrix composites are shown in Figure 11. Under starved oil lubrication, the limited supply of lubricant oil only permits the formation of a discontinuous adsorbed boundary lubrication film on the sliding interface; consequently, the tribological properties of the PEEK composite are determined by the combined influence of both boundary lubrication and localized dry friction. Owing to the inherently poor self-lubricating capability of pure PEEK, its COF exhibits substantial fluctuations and irregular wear, characterized by heterogeneous material removal and surface cracking with the incorporation of h-BN. The fluctuation of the friction coefficient is reduced owing to the self-lubricating properties of h-BN. Meanwhile, a slight increase in the friction coefficient is observed because of the higher surface roughness of PEEK/h-BN/SCFs. It should also be noted that both PEEK/h-BN and PEEK/h-BN/SCFs possess lower oil binding ability than pure PEEK, which reduces the formation efficiency of the boundary lubricant film, so the lubricating performance of the oil cannot be fully utilized. As a result, the wear resistance of PEEK/h-BN/SCFs does not achieve a significant improvement compared with pure PEEK.

## 4. Conclusions

In this study, PEEK composites reinforced with h-BN and SCFs were fabricated via hot pressing. The surface wettability and tribological performance under dry sliding and starved oil lubrication conditions were systematically investigated. Underlying mechanisms were illustrated based on morphological, mechanical and surface wettability analyses. The principal conclusions are drawn as follows:

(1) The incorporation of h-BN and SCFs effectively enhanced the Vickers hardness of the PEEK matrix by constraining the plastic deformation of polymer chains, but concurrently reduces surface oil wettability. This reduction was attributed to the intrinsically low surface energy of both the fillers and the formation of heterogeneous micro–nano surface structures that trap microscopic air pockets at the contact interface.
(2) Under dry sliding conditions, h-BN significantly reduced the friction coefficient through its solid lubrication and heat dissipation effects. The COF reduced by 31.5% compared with pure PEEK. Meanwhile, SCFs further enhance the load-bearing capacity of the matrix and resistance to plastic deformation. The PEEK/h-BN/SCF composite achieved the optimal specific wear resistance, which is 74.6% lower than that of pure PEEK, demonstrating a synergistic anti-wear effect. The dominant wear mechanism transforms from severe adhesive wear and plastic deformation for pure PEEK to mild abrasive wear and fatigue wear.
(3) Under starved oil lubrication, pure PEEK exhibited non-uniform wear characteristics. PEEK/h-BN delivered the lowest and most stable friction performance, whereas SCFs induced a COF increase attributable to elevated surface roughness, while enhancing wear resistance owing to the improved load support and fatigue resistance. The tribological behavior under starved oil conditions was the combined effect of boundary lubrication and solid lubrication, and the reduced oil wettability of composites partially limited the full exertion of oil lubrication efficiency.

In conclusion, the incorporation of h-BN and SCFs as a reinforcing phase effectively improved the tribological properties and wear resistance of PEEK-based composites under dry and starved oil lubrications, affirming their viability as sliding materials under severe operating conditions.

## Figures and Tables

**Table 1:** Chemical composition of the PEEK composites (wt.%).

Materials	PEEK	h-BN	SCFs
Pure PEEK	100	0	0
PEEK/h-BN	90	10	0
PEEK/h-BN/SCFs	85	10	5

## Data Availability

The original contributions presented in this study are included in the article. Further inquiries can be directed to the corresponding authors.
